# Phenotypic profiling of small molecules using cell painting assay in HCT116 colorectal cancer cells

**DOI:** 10.1371/journal.pone.0334025

**Published:** 2025-10-29

**Authors:** Velemir Lavrinenko, Ilona Donskaya, Vladimir Popov, Ekaterina Litau, Varvara Petrova, Stanislav Tyazhelnikov

**Affiliations:** 1 JSC BIOCAD, Saint Petersburg, Russia; 2 St. Petersburg Academic University, St. Petersburg, Russian Federation; University of Chicago, UNITED STATES OF AMERICA

## Abstract

Understanding how small molecules affect cellular morphology is essential for exploring their mechanisms of action (MoA) and identifying new therapeutic candidates. In this study, the Cell Painting Assay (CPA) was used to profile 196 small molecules in HCT116 colorectal cancer cells. By applying t-distributed stochastic neighbor embedding (t-SNE) followed by density-based clustering, 18 distinct phenotypic clusters were identified based on similarities in the quantitative morphological profiles generated from Cell Painting data. Although it was initially hypothesized that clustering would reflect known MoAs, most clusters showed only partial overlap with target-based classifications. Instead, compounds from different MoA classes converged on similar cellular phenotypes, suggesting common downstream effects or shared stress responses. Notably, compounds affecting DNA replication, mitosis, or transcriptional control appeared across multiple clusters, indicating functional diversity within morphologically similar groups. Clusters enriched with mTOR/PI3K inhibitors, spindle poisons, or transcriptional CDK blockers exhibited well-defined phenotypes, supporting the robustness of the assay. In contrast, compounds inducing more subtle phenotypes formed distinct micro-clusters, highlighting the method’s sensitivity. Overall, this study demonstrates that CPA can capture convergent phenotypic signatures that extend beyond target-based classification. These findings underscore the value of phenotype-driven screening for the functional annotation of chemical compounds and may help uncover unexpected relationships among molecules with diverse biological activities.

## Introduction

High-throughput imaging and automated image analysis have become essential tools for studying cellular functions and responses to various treatments. One widely used method in both academic and industrial laboratories is the Cell Painting Assay (CPA) [1]. This technique is particularly effective for investigating how small molecules influence cell morphology. By applying fluorescent dyes to stain different cellular compartments, CPA generates detailed information on cellular structure and responses, making it a valuable tool in drug discovery, toxicology, and disease biology [2]. CPA produces multiparametric profiles by staining multiple subcellular structures, including the nucleus, nucleoli, mitochondria, endoplasmic reticulum, Golgi apparatus, plasma membrane, actin cytoskeleton, and RNA. It utilizes five fluorescent channels to visualize these components within a single cell, with each channel yielding hundreds of features that together form a comprehensive morphological profile. The assay is particularly suitable for high-throughput screening of compound libraries due to its relatively low cost, ease of implementation, and flexibility. Unlike antibody-based staining methods, CPA relies on chemical dyes, making it cost-effective for large-scale studies [3]. Furthermore, the assay has been adapted for diverse biological applications, including mutation profiling in cancer, toxicity assessment of environmental chemicals, and identification of potential treatments for diseases such as COVID-19 [4]. Researchers have also optimized the protocol to simplify, accelerate, and reduce the cost of the assay without compromising image quality or analytical robustness.

The Cell Painting Assay is a phenotypic screening method that differs in several ways from traditional target-based approaches. It enables unbiased discovery, as it does not require prior knowledge of molecular targets, allowing researchers to identify compounds that act through novel mechanisms. Additionally, CPA can reveal multi-target agents, which are particularly valuable for treating complex diseases such as cancer [5]. By capturing the full cellular response, phenotypic screening can uncover both therapeutic effects and potential off-target activities, thereby supporting the selection of safer drug candidates. However, this approach also presents certain challenges. The interpretation of data is often difficult, as high-content screening generates large image datasets that require advanced computational tools for analysis [6]. Moreover, reproducibility may vary, as the results are sensitive to specific experimental conditions.

In this study, the CPA was used to evaluate the effects of 196 commercially available small molecules on the HCT116 cell line, a well-established model for colorectal cancer research. Although CPA has been successfully applied to various cancer cell lines, such as A549, U2OS, and MCF-7 [3], its use in colorectal cancer models remains limited. Most previous studies have focused on general cytotoxicity or mechanistic profiling in non-colorectal contexts. This gap underscores the need for a systematic evaluation of CPA’s utility in colorectal cancer, one of the most common and lethal malignancies worldwide, driven by a combination of genetic mutations and environmental factors [7]. HCT116 cells, derived from human colorectal adenocarcinoma, are widely used in cancer research due to their rapid proliferation, high survival rate, and ability to form colonies. This cell line harbors key mutations in major signaling pathways, including Wnt/β-catenin, PI3K/AKT/mTOR, and MAPK [8–10], which are critical for regulating cell growth, survival, and metastasis. These characteristics make HCT116 cells a suitable model for studying colorectal cancer and evaluating the effects of various drugs. Moreover, they are sensitive to several commonly used chemotherapeutic agents, such as 5-fluorouracil, doxorubicin, and paclitaxel [11–13], which are standard treatments for colorectal cancer.

The Cell Painting Assay enables the generation of detailed phenotypic profiles by capturing hundreds of cellular features following treatment with each compound. By comparing these profiles across the 196 tested compounds, it becomes possible to identify patterns associated with specific mechanisms of action. The aim of this study is to evaluate whether CPA can serve as a reliable approach for distinguishing the cellular effects of different compounds on the HCT116 colorectal cancer cell line and to provide deeper insights into how commercially available molecules act at the cellular level.

## Materials and methods

The HCT116 colorectal cancer cell line was obtained from the American Type Culture Collection (ATCC) and maintained according to the manufacturer’s instructions in DMEM medium (Gibco, 12-100-061), supplemented with 10% heat-inactivated fetal bovine serum (FBS) (Gibco, 16140−071) and 2 mM L-glutamine (PanEco, F032). For all experiments, cells were cultured up to the 20th passage to ensure consistency and reliability of the results.

A total of 196 small molecules have been applied with the only purpose of testing their effects on the cells in the laboratory. All compounds were commercially available and used to test the assay’s ability to identify mechanisms of action. The selection of tested compounds was aimed at covering a broad range of known mechanisms of action, including kinase inhibitors, DNA-damaging agents, metabolic regulators, and other functional classes. The primary goal was to include well-characterized compounds with activity in human cancer cells. In addition, priority was given to molecules expected to induce strong and diverse morphological changes, in order to maximize the phenotypic variation captured by the assay. The molecules were divided into eight cohorts, each comprising 29 compounds (S1 Fig in S1 File). Alpelisib and KU-0063794 were included as positive controls. Alpelisib, a PI3Kα inhibitor and component of the PI3K/AKT/mTOR pathway, is commonly used as a reference compound in morphological profiling studies. KU-0063794 inhibits both mTORC1 and mTORC2 and is known to produce clear and reproducible responses in CPA. These control compounds were selected to evaluate the sensitivity of the analysis pipeline and to compare clustering consistency.

The Cell Painting Assay was performed following the protocol described by Cimini et al., 2023 [4], including key steps such as cell seeding, compound treatment, staining and fixation, image acquisition, and data analysis.

**Fig 1:**
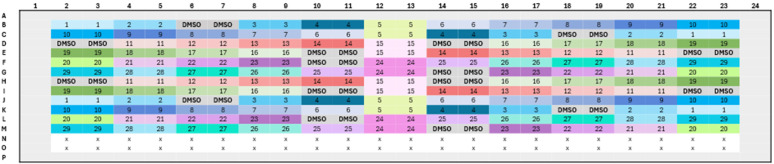
Layout of compound distribution in a 384-well plate used for the Cell Painting Assay.

1. **Cell Culture**: HCT116 cells were cultured as described above. Cells were maintained at a density of 5,000 cells per cm² and passaged twice a week.
2. **Seeding**: A cell suspension was added into 384-well plates at a density of 1,000 cells per well using a multichannel pipette. Plates were then incubated at 37°C in a 5% CO₂ atmosphere for 24 hours.
3. **Compound Addition**: Each well received a test compound at a final concentration of 1 µM, while wells containing DMSO served as negative controls. This concentration was selected based on preliminary dose–response experiments. In these tests, compounds were titrated across a range of concentrations, and 1 µM was found to induce clear morphological changes in most cases without causing excessive cytotoxicity. Therefore, it was chosen as an optimal balance between phenotypic detectability and cell viability for the main screen. Each compound was added to eight distinct wells located in different regions of the 384-well plate, following a manual randomization strategy to minimize positional biases. The plate layout is shown in Fig 1. This manual randomization approach ensured a high degree of randomization, ease of implementation, and reduced the likelihood of pipetting errors.

4 **Compound Incubation**: Cells were incubated with the compounds for 48 hours at 37°C in a 5% CO₂ environment.
5 **Preparation for Staining and Permeabilization**: Two distinct staining solutions were prepared. Solution 1 consisted of DMEM medium without FBS and PhenoVue 641 Mitochondrial Stain. Solution 2 was composed of 1 × diluent, 10% Triton X-100, PhenoVue Fluor Hoechst 33342 Nuclear Stain, PhenoVue 512 Nucleic Acid Stain, PhenoVue Fluor 488–Concanavalin A, PhenoVue Fluor 555–WGA, and PhenoVue Fluor 568–Phalloidin. The volumes and concentrations of each component were determined according to the manufacturer’s instructions. Table 1 provides details on the specific dyes used for imaging various cellular structures.

**Table 1:** Staining reagents and their corresponding cellular compartments visualized in the Cell Painting Assay.

Dye	Cellular Compartment
PhenoVue 641 Mitochondrial Stain	Mitochondria
PhenoVue Fluor 555 – WGA	Actin, plasma membrane
PhenoVue Fluor 488 – Concanavalin A	endoplasmic reticulum
PhenoVue Fluor 568 – Phalloidin	Actin, cytoskeleton
PhenoVue Fluor Hoechst 33342 Nuclear Stain	Nucleus
PhenoVue 512 Nucleic Acid Stain	Nucleic acids

6 **Mitochondrial Staining**: The culture medium was partially aspirated, after which Solution 1 was added to each well. The plates were then incubated at 37 °C for 30 minutes in the dark.
7 **Fixation**: After aspiration, the cells were fixed with BD Cytofix and incubated for 20 minutes at room temperature in the dark.
8 **Washing**: The cells were washed twice with HBSS, followed by complete removal of the washing solution.
9 **Permeabilization and Staining**: Solution 2 was added to the wells, and the cells were incubated for 30 minutes at room temperature in the dark.
10 **Final Wash and Storage**: The plates were washed twice with HBSS. For long-term storage at 4 °C, the wells were filled with HBSS containing 0.05% NaN₃.
11 **Imaging**: Imaging was performed using the CellInsight™ CX7 LED Pro High-Content Screening (HCS) Platform (Thermo Fisher Scientific) at 20 × magnification. Exposure times were manually optimized for each fluorescence channel to ensure sufficient signal intensity while minimizing overexposure: Hoechst (0.0135 s), phalloidin/WGA (0.0172 s), MitoTracker (0.123 s), ER Tracker (0.0123 s), nucleic acid stain (0.0455 s), and brightfield (0.0018 s). Imaging was performed in widefield mode using a 2 × 2 binning configuration with fixed exposure settings. Background correction was applied across all channels.
12 **Image Analysis**: Images were analyzed using CellProfiler, with the analysis divided into three main stages: (i) illumination correction, (ii) quality control (QC), and (iii) morphological feature extraction. The entire image analysis procedure was performed according to the protocol described by Garcia-Fossa et al., 2023 [14].
13 **Construction of Similarity Matrix**: Pearson correlation analysis was performed to assess the relationships between morphological features obtained from the Cell Painting Assay. A correlation matrix was generated and visualized using Morpheus software (https://software.broadinstitute.org/morpheus/). Raw morphological data obtained from image analysis were uploaded to the platform, and Pearson correlation coefficients were calculated for all pairwise feature comparisons. A heatmap was generated to highlight patterns of association.
14 **T-SNE Analysis**: To visualize the high-dimensional phenotypic data, t-distributed Stochastic Neighbor Embedding (t-SNE) analysis was performed using Python’s Scikit-learn library (v1.5.1). This analysis enabled the projection of morphological profiles obtained from the CPA into two dimensions, facilitating the identification of similarities and differences between compounds. The algorithm was run with various parameter combinations, including perplexity (3–50), learning rate (50–1000), and number of iterations (500–3000), to explore their effects on clustering outcomes. In addition, morphological features were aggregated by compound using the median and subsequently scaled with the StandardScaler function. This normalization step was particularly important for the positive controls, which were included in every compound cohort during the experiment.

The resulting two-dimensional representations were subsequently clustered using the Density-Based Spatial Clustering of Applications with Noise (DBSCAN) algorithm implemented in Scikit-learn (v1.5.1). Key DBSCAN parameters, such as epsilon (ε) and the minimum number of samples per cluster (minPts), were systematically varied to assess their influence on clustering results.

## Results

Following the completion of the Cell Painting Assay, the morphological effects of treatment with 196 compounds on the HCT116 cell line were analyzed. As part of the analysis, a correlation matrix was generated using Pearson correlation coefficients. The matrix (S2 Fig in S1 File) provides insight into the degree of similarity between the morphological profiles induced by each compound. Higher correlation values indicate compounds that elicit similar phenotypic effects, which may suggest a shared mechanism of action or involvement in related target pathways. In contrast, low or negative correlations reflect distinct or opposing effects, underscoring the diversity of compound responses within the dataset.

t-SNE analysis was conducted to visualize the data and evaluate whether the tested compounds could be clustered based on their morphological effects on cells. Given that the choice of parameters in dimensionality reduction and clustering can significantly affect the results, a systematic grid search was performed to explore multiple parameter combinations. In total, 288 configurations were tested, varying perplexity, learning rate, and number of iterations for t-SNE, as well as epsilon (ε) and the minimum number of samples (minPts) for the DBSCAN algorithm.

**Fig 2:**
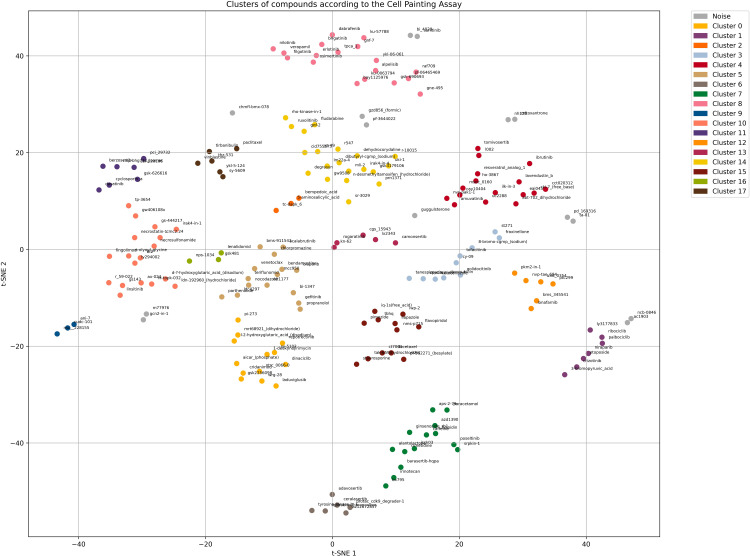
Two-dimensional t-SNE projection of the morphological profiles of HCT116 cells treated by tested compounds. Clustering was performed using the DBSCAN algorithm (eps = 5, min_samples = 3) after dimensionality reduction (perplexity = 5, learning rate = 100, n_iter = 1000). Each point represents one compound; gray points indicate objects identified as noise. Clusters are visually distinguished by color. The resulting silhouette score is 0.4.

Among the tested configurations, the parameter set with perplexity = 5, learning rate = 100, n_iter = 1000, ε = 5, and minPts = 3 consistently produced well-defined and clearly separated clusters with minimal overlap (silhouette score = 0.4). Using this setup, the compounds were grouped into 18 distinct clusters, while 14 profiles could not be assigned to any cluster and were labeled as “Noise” (Fig 2). Notably, the positive controls Alpelisib and KU-0063794 exhibited very high morphological similarity and consistently clustered together, supporting the stability and reliability of the analytical pipeline.

Based on the dot plot generated from the t-SNE analysis, all 196 compounds were represented as individual points, with each point corresponding to a single compound. The primary objective following this visualization was to identify and interpret phenotypic clusters, which was successfully achieved. Table 2 summarizes the functional theme of each cluster along with the number of compounds it contains. S3 Fig in S1 File provides the detailed composition of each cluster, listing the compounds grouped according to their phenotypic profiles. To support the morphological interpretation of the identified clusters, representative microscopy images of HCT116 cells treated with compounds from each cluster are presented in S4 Fig in S1 File.

**Table 2:** Summary of functional themes assigned to each cluster, along with the number of compounds grouped within each cluster.

Cluster	Amount of compounds	Functional theme
0	13	Metabolic + checkpoint stress
1	7	DNA damage/ replication checkpoint
2	3	AMPK/ NF-κB brake
3	10	Proteotoxic + inflammasome stress
4	16	RTK/WNT off + translation halt
5	15	Mitotic catastrophe + NF-κB off
6	6	Checkpoint override & Pol II pause
7	14	ATR/Aurora stress + inflammasome brake
8	18	PI3K–Akt–mTOR pause
9	3	Spindle poison + HDAC/CREB
10	18	Lipid-signaling pause + necro brake
11	7	Cytoskeleton collapse + ATR stress
12	6	NF-κB/ RAS brake
13	5	Purine/Ca² ⁺ blebbing
14	20	Second-messenger & actin rewiring
15	12	Spindle arrest + WNT off
16	3	Low-stress dampening
17	6	Spindle poisons + CDK block

Despite the initial hypothesis that phenotypic similarity would correspond to a shared MoA, the clustering results revealed a more complex organization. While certain clusters contained compounds with similar known activities, the overall structure indicated morphological convergence rather than a strict correspondence to MoA.

The distribution of compounds across clusters was uneven, ranging from three to twenty compounds per cluster. Cluster 14 was the largest, comprising 20 compounds and functionally associated with second messenger signaling and actin cytoskeleton reorganization. This cluster included compounds such as CID755673 [15], degrasyn [16], and dehydrocorydaline [17]. Clusters 8 and 10 were of similar size, each containing 18 compounds. Cluster 8 represented phenotypes linked to suppression of the PI3K–Akt–mTOR pathway (e.g., alpelisib [18], brigatinib [19]), while cluster 10 was associated with alterations in lipid signaling and necroptosis (e.g., fingolimod [20], linsitinib [21], necrostatin-1 [22]). In contrast, clusters 2, 9, and 16 each contained only three compounds, which may reflect either highly specific phenotypes or responses below the threshold of reliable detection.

Importantly, functionally related compounds are not always grouped within the same cluster, suggesting that different molecular pathways may converge on similar phenotypic outcomes. This is particularly evident in the case of mitotic disruption: compounds known to interfere with cell division appear in clusters 5, 9, 15, and 17. Cluster 5 is enriched with molecules that induce mitotic catastrophe along with suppression of NF-κB signaling (e.g., acalabrutinib [23], bendamustine [24]), while cluster 17 includes spindle poisons and CDK inhibitors such as paclitaxel [25], SY-5609 [26], and THZ-531 [27], all of which are known to cause prometaphase arrest. Cluster 15 represents a hybrid phenotype combining spindle checkpoint activation and WNT pathway inhibition (e.g., docetaxel [28], CT7001 [29], flavopiridol [30]). These findings highlight the complexity of compound-induced phenotypes and suggest that some molecules may exert additional, previously unreported effects on unexpected targets. Such off-target or pleiotropic activities may contribute to the observed phenotypic patterns and underscore the value of unbiased morphological profiling in revealing hidden biological activities.

Compounds affecting DNA replication and the DNA damage response also exhibit distributed clustering, further emphasizing the complexity of phenotypic relationships. Cluster 1 includes inhibitors of the replication checkpoint, such as etoposide [31], niraparib [32], and the glycolysis inhibitor 3-bromopyruvic acid [33]. In contrast, cluster 6 is characterized by checkpoint override and transcriptional pausing and contains compounds such as adavosertib [34] and ceralasertib [35].

Clusters associated with cytoskeletal disruption and changes in cell adhesion also demonstrate phenotypic convergence. Cluster 11 captures cells undergoing cytoskeletal collapse and ATR-mediated stress, including compounds such as berzosertib [36], dasatinib [37], and cyclosporin A [38]. Cluster 13 is characterized by blebbing phenotypes associated with purine signaling, represented by camonsertib [39], KN-62 [40], and CGS-15943 [41]. In contrast, cluster 0 reflects a broader phenotype related to metabolic and checkpoint stress and includes compounds such as 1-deoxynojirimycin [42] and AICAR [43].

Interestingly, several clusters combine seemingly unrelated pathways yet produce coherent phenotypic profiles. For example, cluster 3 merges proteotoxic stress with inflammasome activity (golidocitinib [44], doxorubicin [12], CY-09 [45]), while cluster 7 reflects a combination of ATR inhibition and immune-related inflammasome suppression (alantolactone [46], APS-2–79 [47], AZD1390 [48]). In addition, a group of compounds was classified as “Noise” due to either inconsistent replicate behavior or weak phenotypic effects. This group included AC1903 [49], BI-4020 [50], and CHMFL-BMX-078 [51], and likely reflects off-target effects, edge-related artifacts, or low-potency phenotypes.

Overall, these results illustrate that phenotypic clustering based on the Cell Painting Assay does not always align with mechanistic classifications but instead reveals biologically meaningful groupings driven by convergence in cellular morphology. The observed clusters constitute a phenotypic map of how HCT116 cells respond to diverse chemical perturbations and may provide a framework for identifying novel compound activities based on phenotypic profile similarity rather than mechanism of action alone.

## Discussion

This study demonstrates the utility of the Cell Painting Assay for phenotypic screening in the context of colorectal cancer research. By applying CPA to the HCT116 colon cancer cell line, we characterized the morphological effects of 196 commercially available compounds and identified distinct clusters based on their phenotypic profiles. These clusters suggest interactions involving related or overlapping signaling pathways and mechanisms of action. Overall, the findings highlight not only the assay’s capacity to distinguish between compounds, but also its broader applicability in drug discovery and mechanistic investigation.

Clustering divided the compound library into 18 groups, each characterized by a distinct phenotypic fingerprint. Although the compounds within a given cluster may have different primary targets, they were grouped together based on their similar morphological effects on cells. These shared phenotypes often reflected disruptions in DNA replication, inhibition of cell proliferation, or interference with survival signaling pathways. This demonstrates that CPA is capable of capturing functional convergence that may not be detected by traditional single-parameter assays.

At the same time, this study highlights several limitations of the approach. Processing CPA data requires robust computational tools, and even minor variations in fixation or staining protocols can significantly influence the clustering results. The presence of “noise” profiles indicates that some compounds induce unique or non-standard morphological patterns that may not be captured by current similarity metrics or clustering algorithms.

In this study, t-SNE was selected for dimensionality reduction over alternative methods such as UMAP and PCA, due to its ability to preserve local structure within high-dimensional data. This is particularly important for Cell Painting profiles, where subtle phenotypic differences between treatment conditions may reflect biologically meaningful effects. For clustering, the DBSCAN algorithm was chosen instead of methods like K-means, as it does not require predefining the number of clusters and can identify groups of varying shapes and densities. Additionally, DBSCAN effectively labels outliers as noise, which is especially valuable in phenotypic screens, where some compounds induce distinct or non-converging morphological patterns. The combination of t-SNE and DBSCAN provided an unsupervised, data-driven approach to uncover functional groupings within the morphological feature space.

Moreover, although this study focused exclusively on HCT116 cells, expanding the application of CPA to additional colorectal cancer models could provide broader insights into compound specificity and efficacy across diverse genetic backgrounds. Furthermore, integrating orthogonal assays to validate the mechanisms of action of lead compounds identified through CPA would further strengthen the biological relevance and translational potential of the findings.

In summary, this study highlights the potential of the Cell Painting Assay as a powerful tool for phenotypic drug discovery. By enabling high-throughput, morphology-based classification of compounds, CPA facilitates the identification of potential therapeutic candidates and enhances our understanding of complex cellular responses in the context of cancer biology.

## Supporting information

S1 File Contains the supporting figures including cohorts’ composition in the HCT116 Cell Painting Assay, phenotypic similarity matrix of HCT116 cells treated with tested compounds, detailed composition of each cluster and microscopy images illustrating morphological phenotypes of cluster representatives. (DOCX)
