# Supplementary material for: Phenotypic profiling of small molecules using cell painting assay in HCT116 colorectal cancer cells
Source: PLoS One. 2025 Oct 29;20(10):e0334025. doi: 10.1371/journal.pone.0334025 (PMC12571279; doi:10.1371/journal.pone.0334025)

**Supporting information for “Phenotypic Profiling of Small Molecules Using Cell Painting Assay in HCT116 Colorectal Cancer Cells”**

**S1 Fig. Cohorts Composition of Tested Compounds in the HCT116 Cell Painting Assay.**


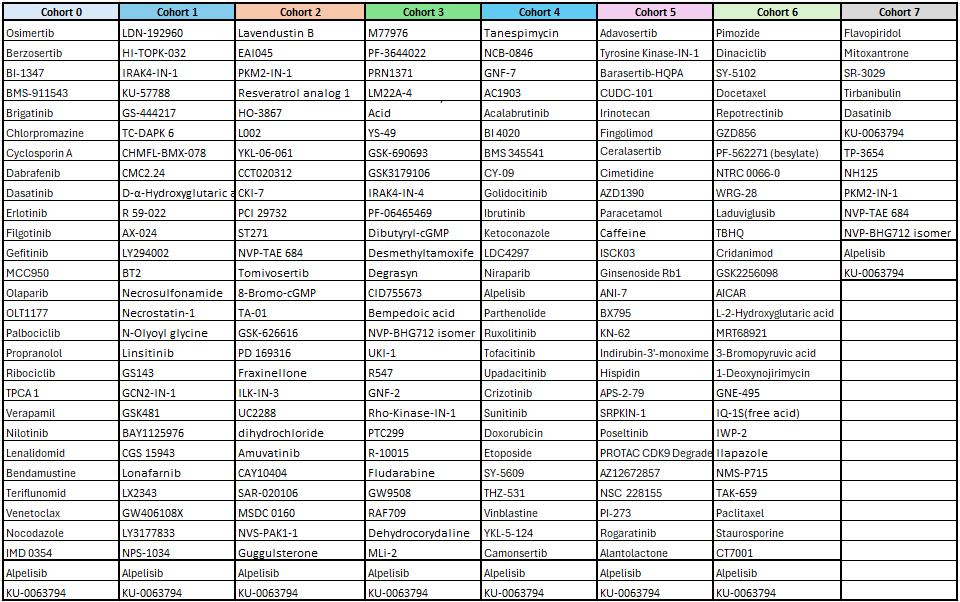


**S2 Fig. Phenotypic Similarity Matrix of HCT116 Cells Treated with Tested Compounds.**


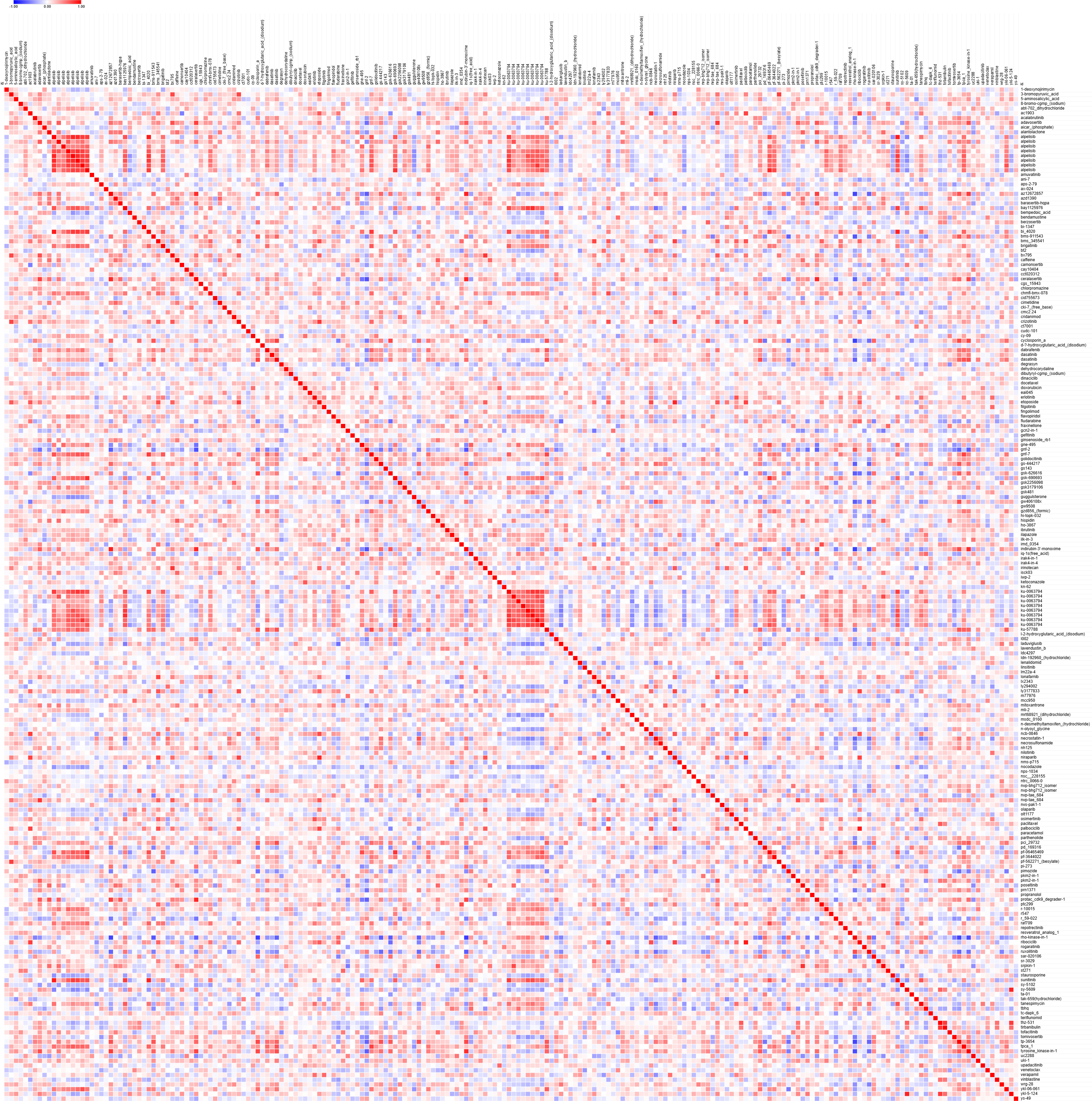


**S3 Fig. Detailed composition of each cluster, listing all compounds grouped within individual clusters.**

**S4 Fig. Microscopy Images Illustrating Morphological Phenotypes of Cluster Representatives.** Scale bar: 25 µm.

**S4.1 Fig. Cluster 0 – Deoxynojirimycin.**

**
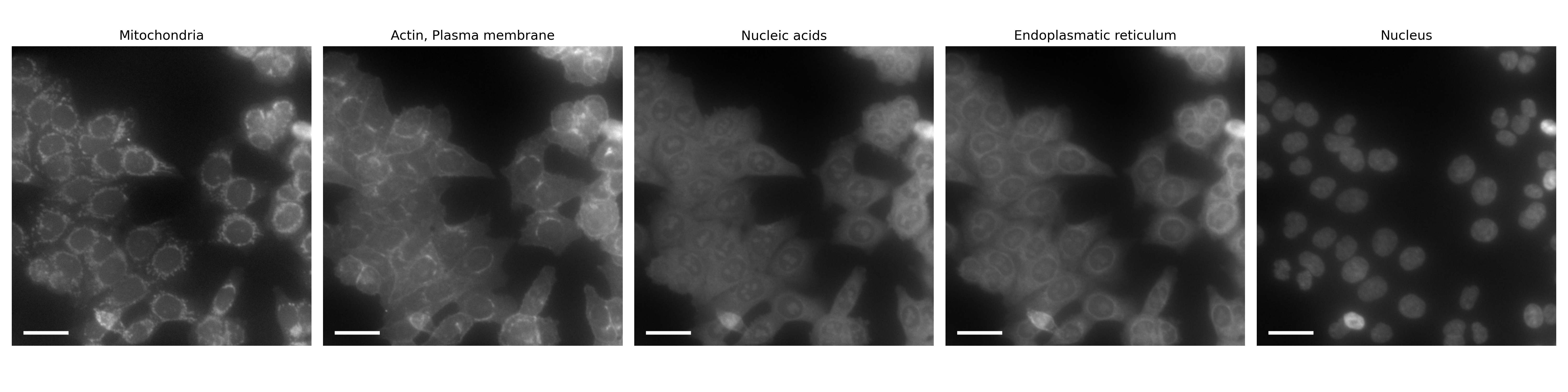
**

**S4.2 Fig. Cluster 1 – Etoposide.**

**
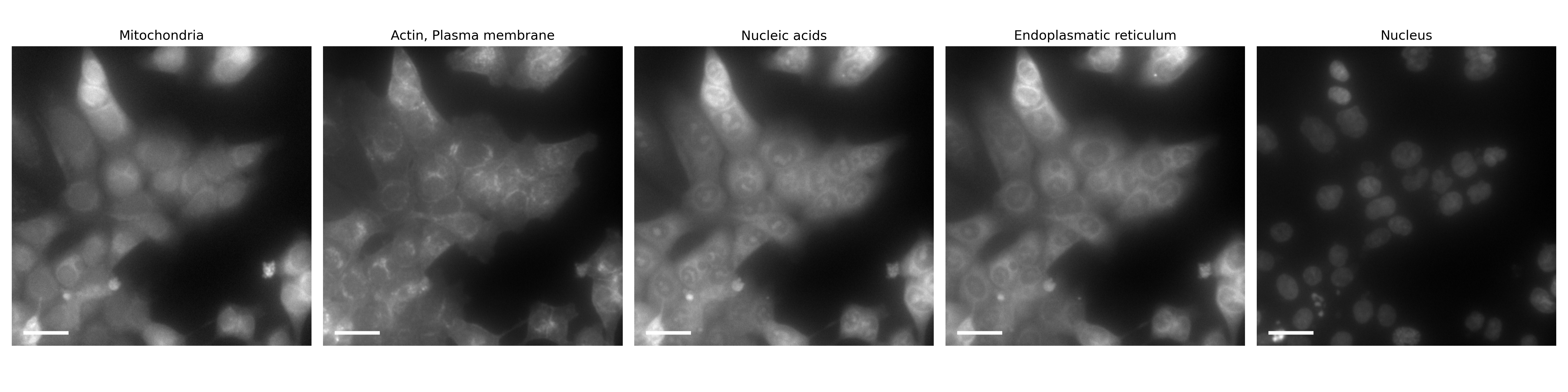
**

**S4.3 Fig. Cluster 2 – Bempedoic acid.**

**
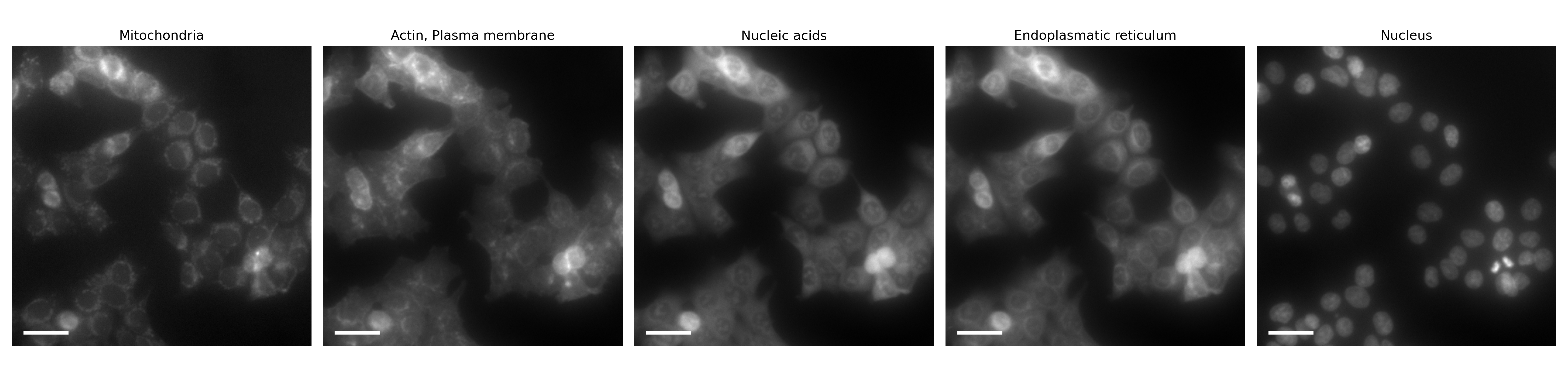
**

**S4.4 Fig. Cluster 3 – Doxorubicin.**

**
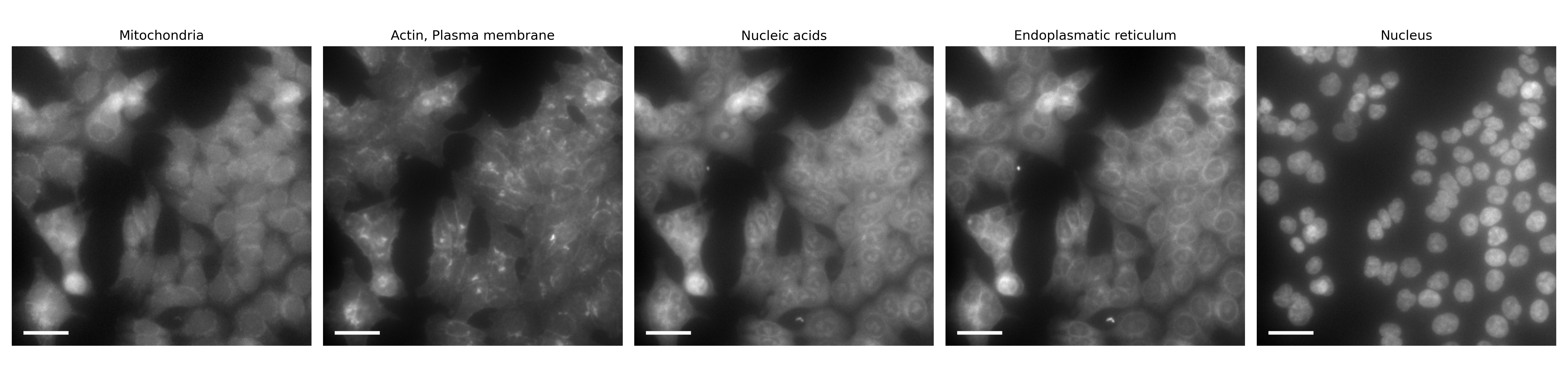
**

**S4.5 Fig. Cluster 4 – Amuvatinib.**

**
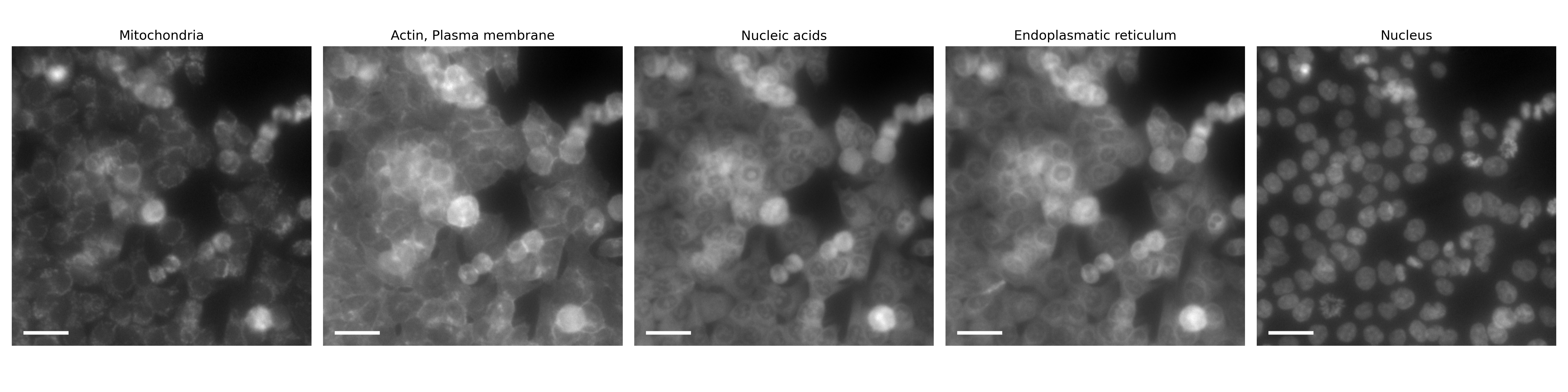
**

**S4.6 Fig. Cluster 5 – Acalabrutinib.**

**
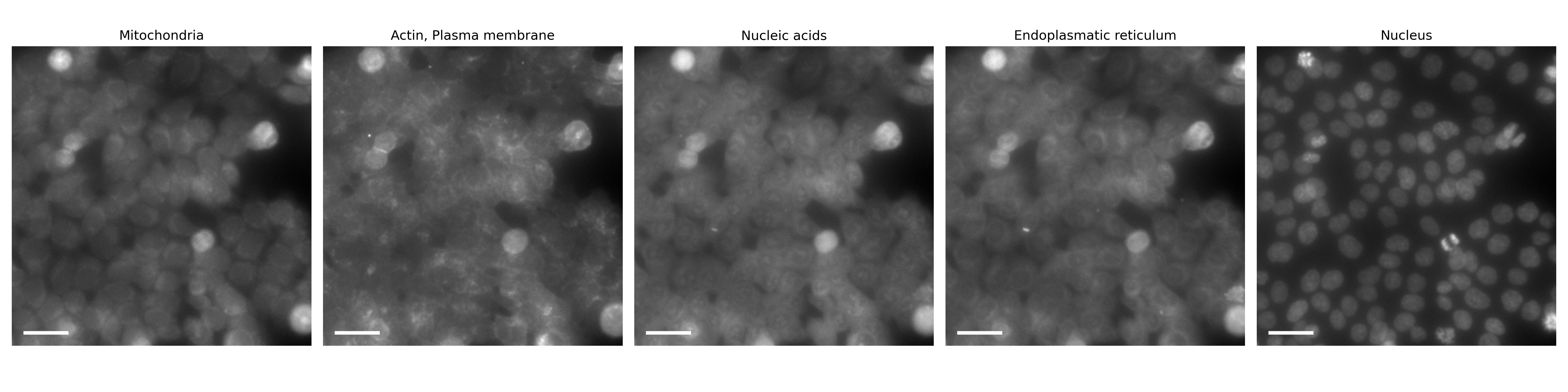
**

**S4.7 Fig. Cluster 6 – Ceralasertib.**

**
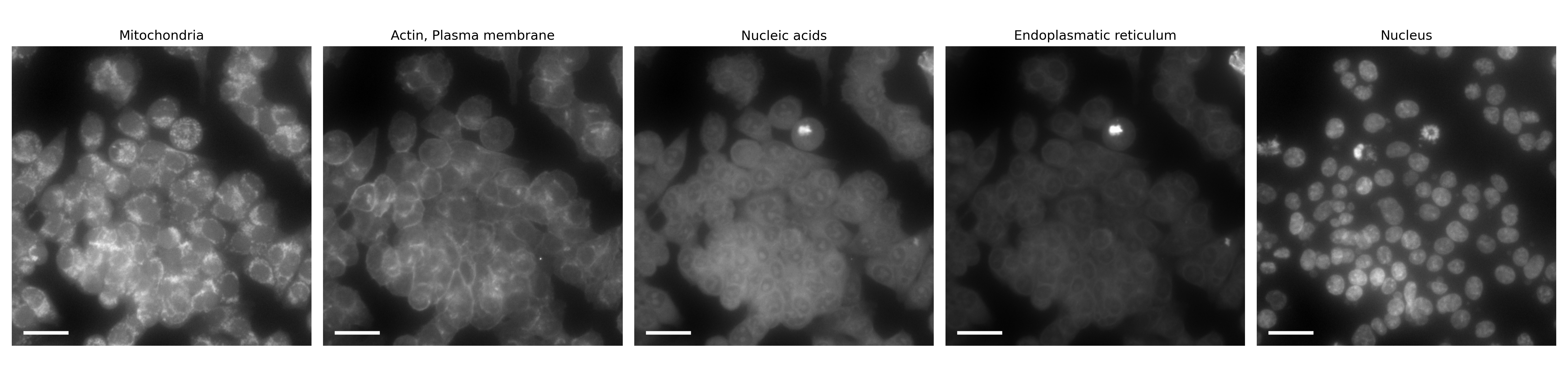
**

**S4.8 Fig. Cluster 7 – Alantolactone.**

**
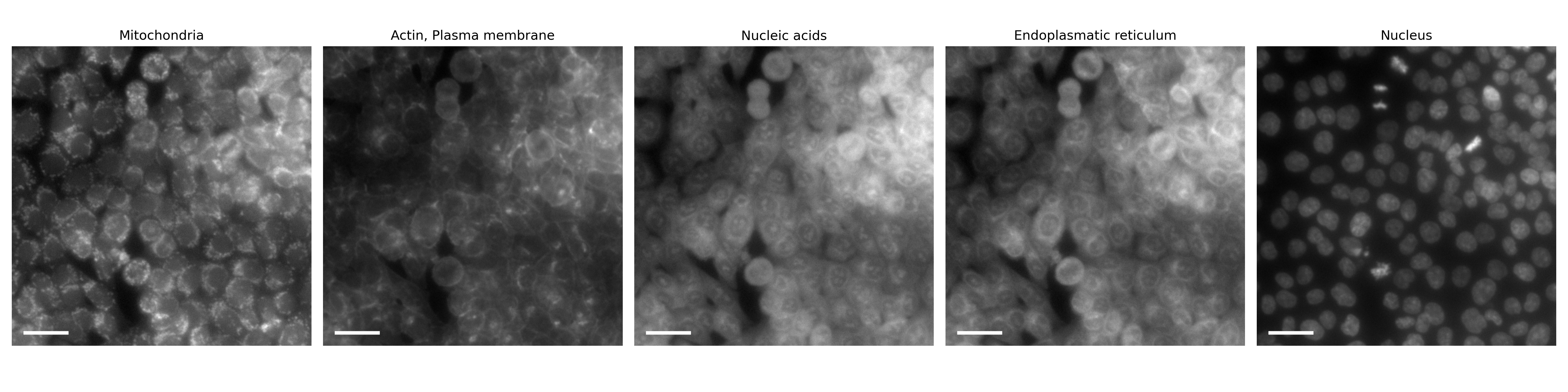
**

**S4.9 Fig. Cluster 8 – Alpelisib.**

**
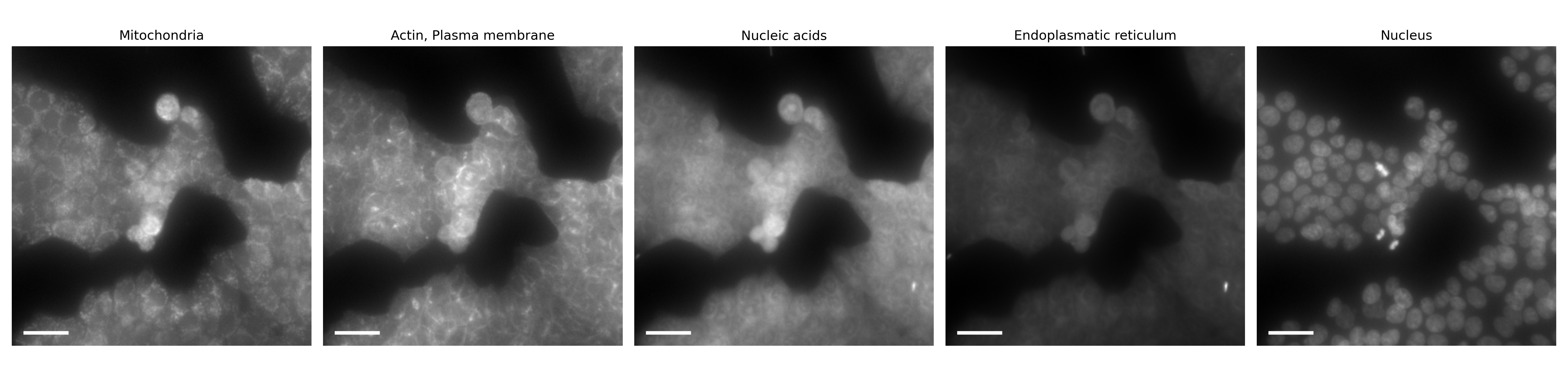
**

**S4.10 Fig. Cluster 8 – KU-0063794.**

**
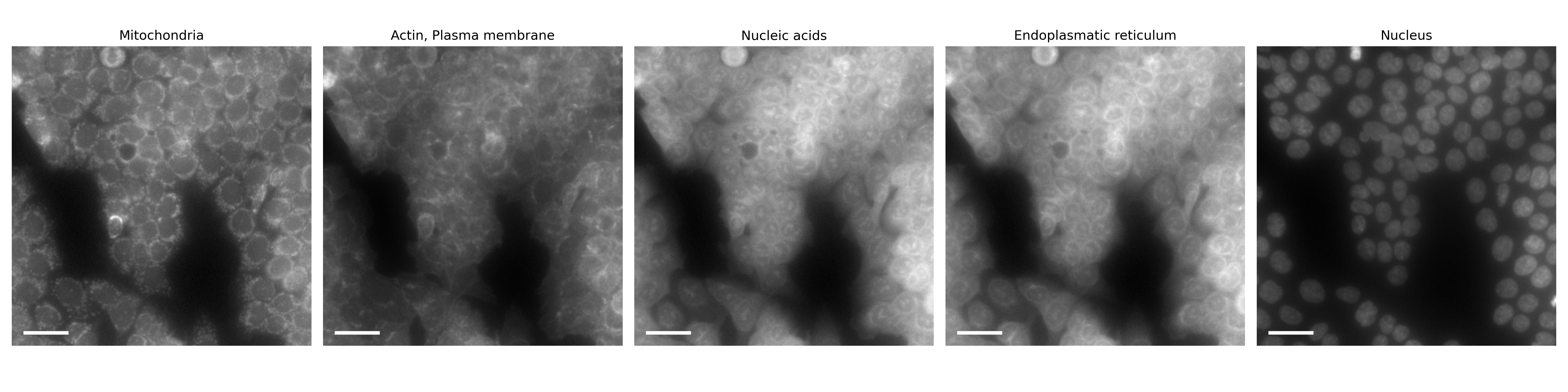
**

**S4.11 Fig. Cluster 9 – Ani 7.**

**
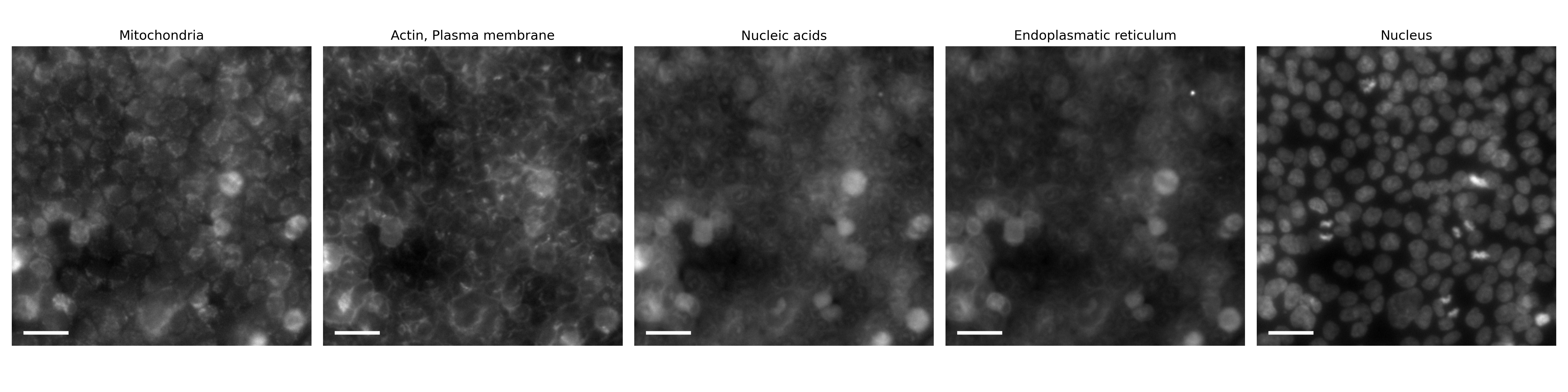
**

**S4.12 Fig. Cluster 10 – Fingolimod.**

**
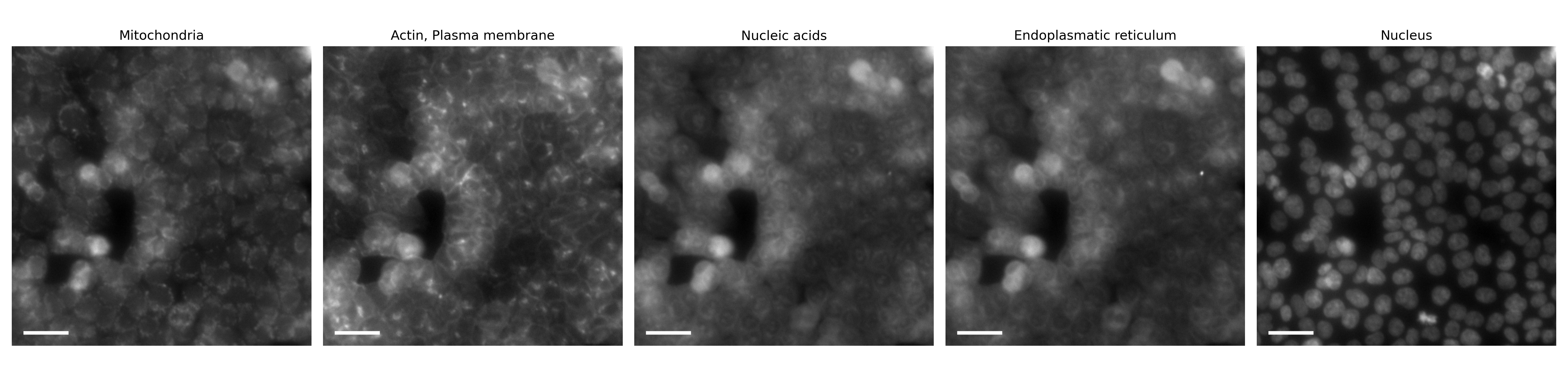
**

**S4.13 Fig. Cluster 11 – Dasatinib.**

**
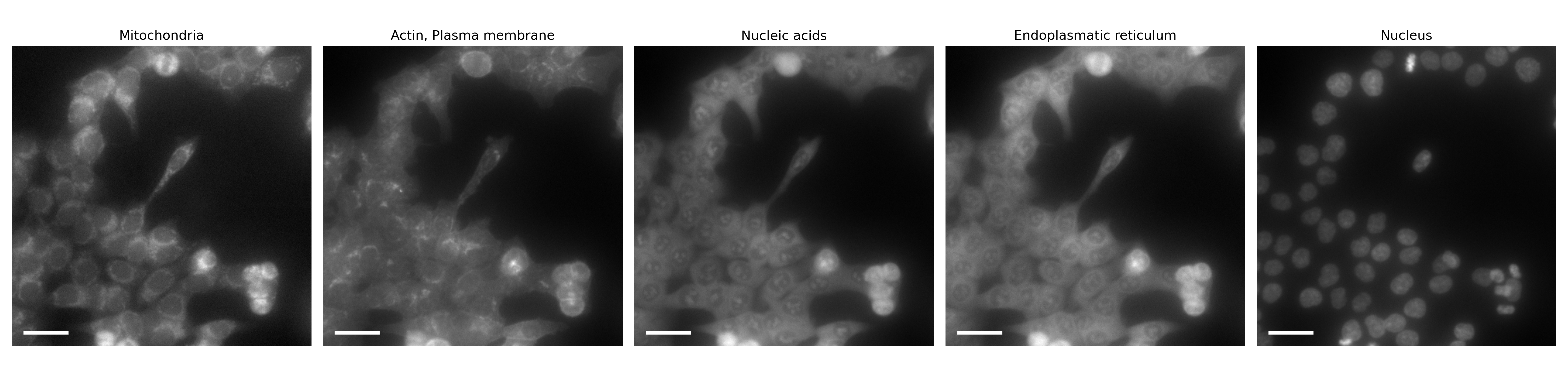
**

**S4.14 Fig. Cluster 12 – Lonafarnib.**

**
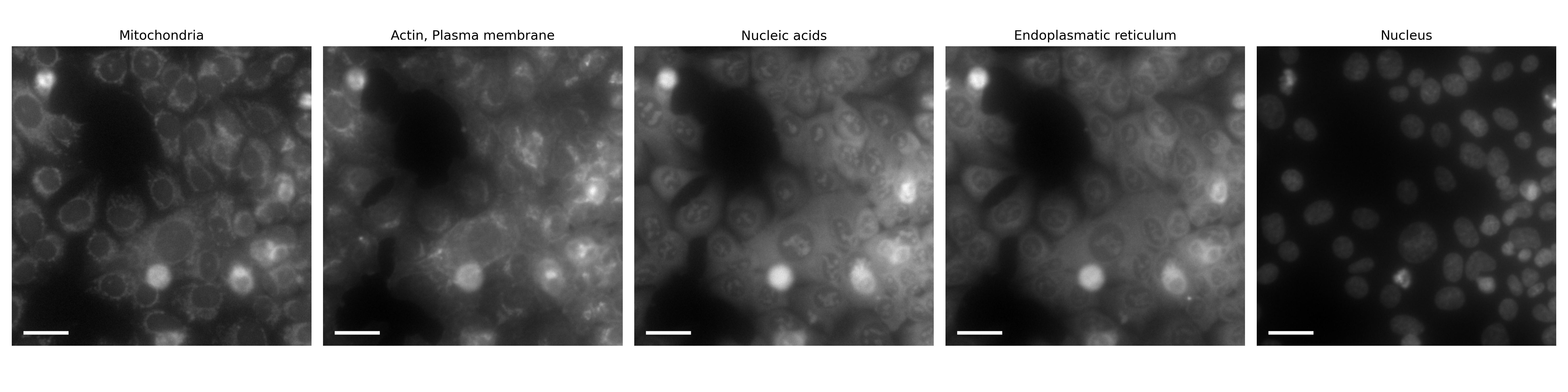
**

**S4.15 Fig. Cluster 13 – Camonsertib.**

**
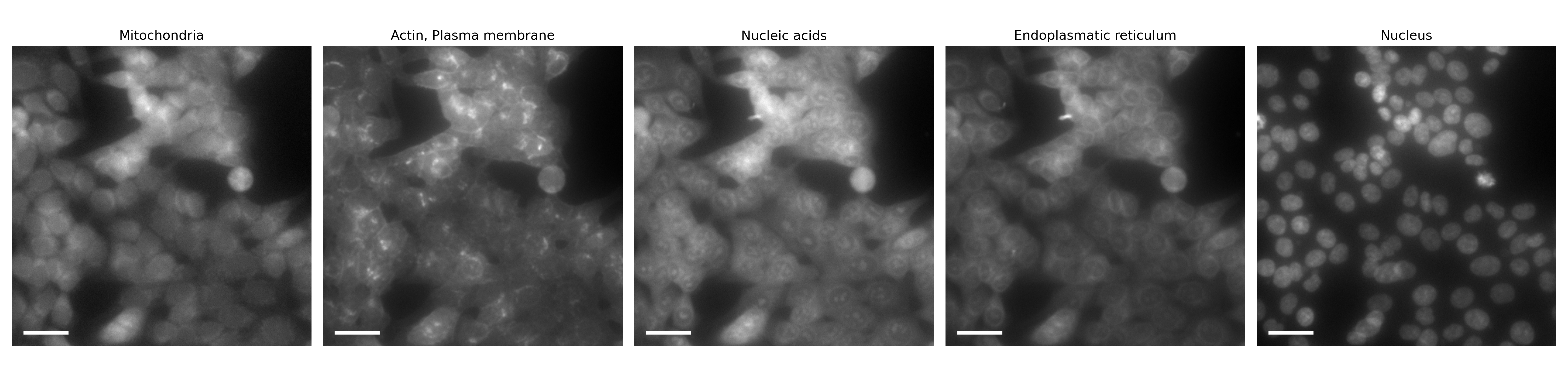
**

**S4.16 Fig. Cluster 14 – Ruxolitinib.**

**
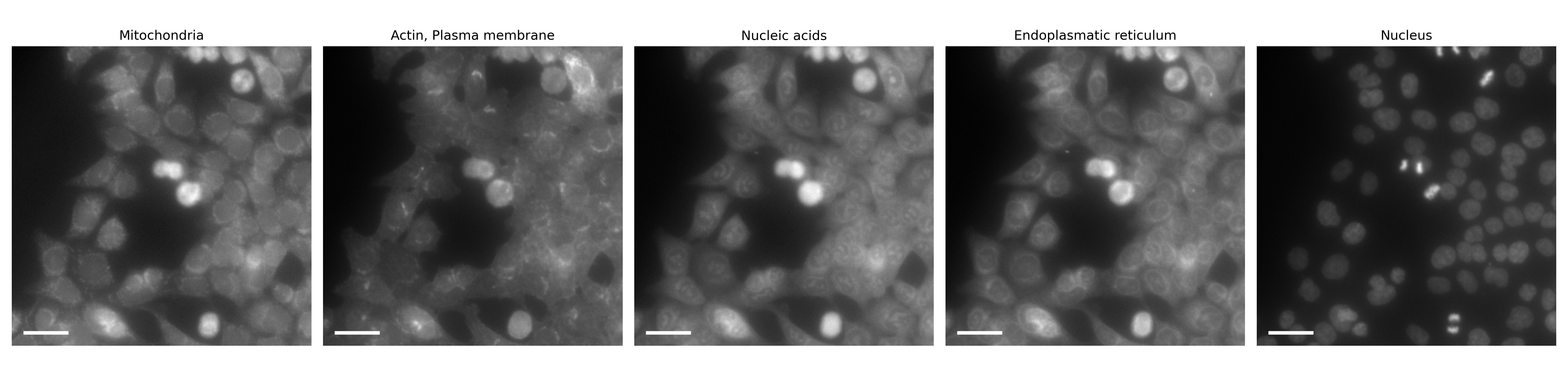
**

**S4.17 Fig. Cluster 15 – Docetaxel.**

**
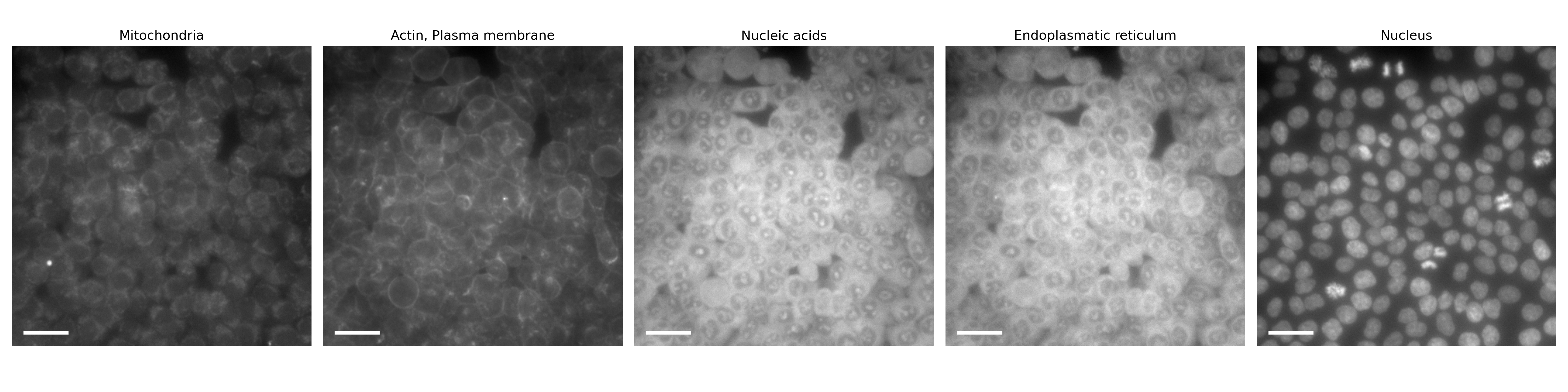
**

**S4.18 Fig. Cluster 16 – Lenalidomid.**

**
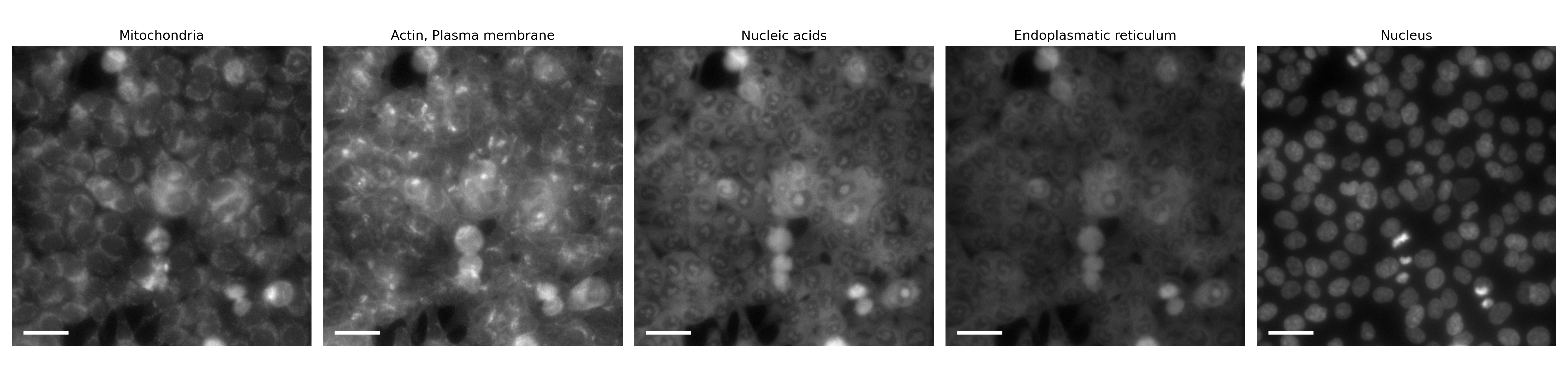
**

**S4.19 Fig. Cluster 17 – Paclitaxel.**

**
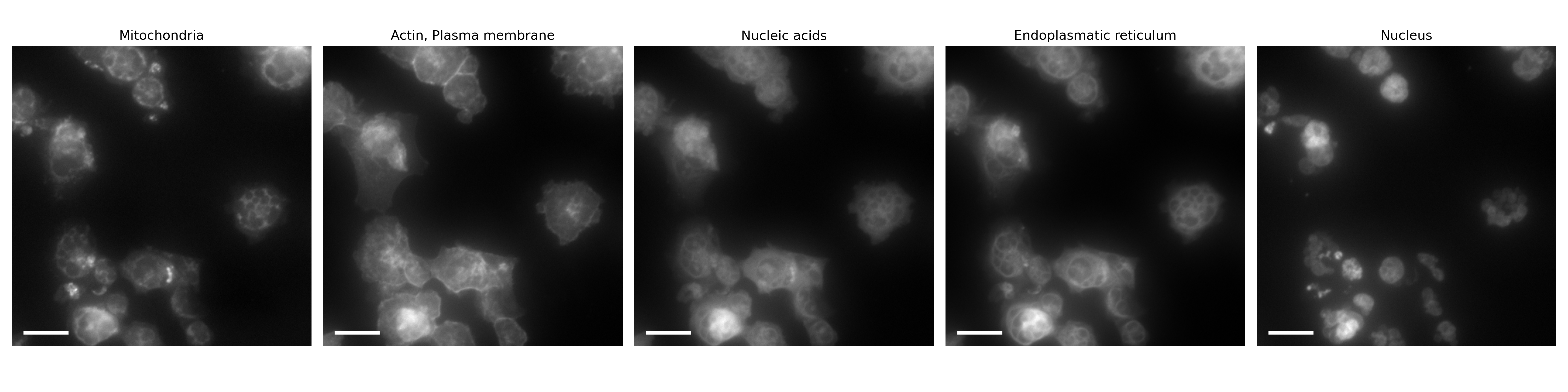
**

**S4.20 Fig. DMSO.**

**
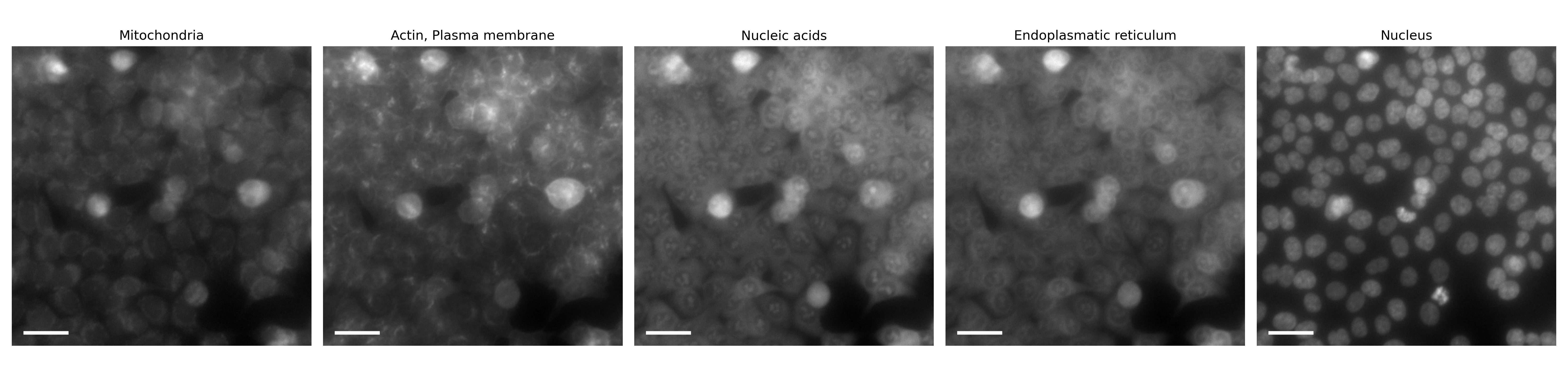
**

**S4.21 Fig. Cluster “Noise” – Sunitinib.**


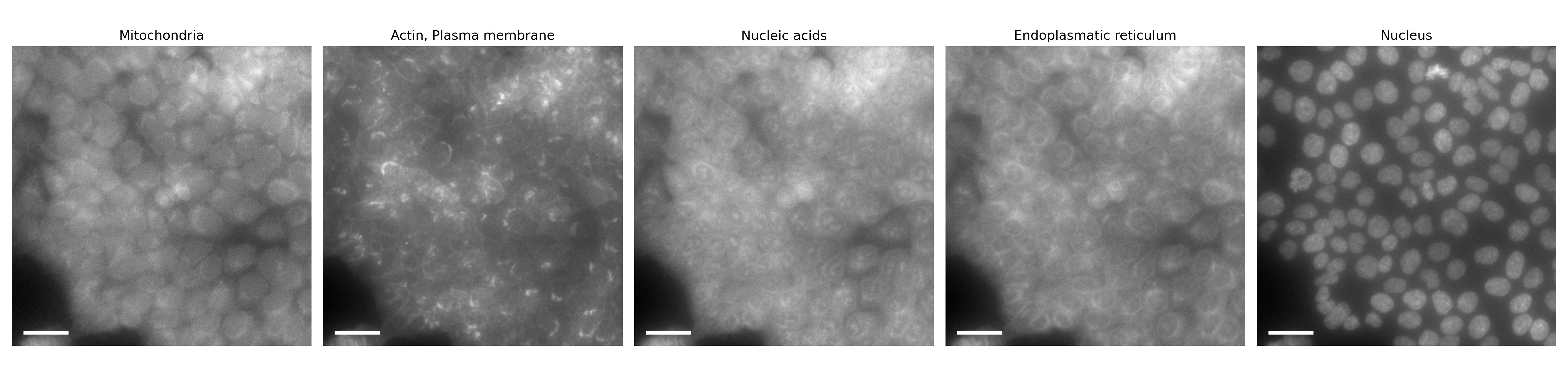

Supplement: S1 File — (DOCX) [file pone.0334025.s001.docx]
